# An improved method for culturing patient-derived colorectal cancer spheroids

**DOI:** 10.18632/oncotarget.25134

**Published:** 2018-04-24

**Authors:** Hiroyuki Miyoshi, Hisatsugu Maekawa, Fumihiko Kakizaki, Tadayoshi Yamaura, Kenji Kawada, Yoshiharu Sakai, M. Mark Taketo

**Affiliations:** ^1^ Division of Experimental Therapeutics, Graduate School of Medicine, Kyoto University, Yoshida-Konoé-cho, Sakyo-ku, Kyoto 606-8501, Japan; ^2^ Department of Surgery, Graduate School of Medicine, Kyoto University, Shogoin-Kawahara-cho, Sakyo-ku, Kyoto 606-8507, Japan; ^3^ Office of Society-Academia Collaboration for Innovation, Kyoto University, Yoshida-Honmachi, Sakyo-ku, Kyoto 606-8501, Japan

**Keywords:** colorectal cancer, spheroid, chemical screening, cancer stem cell, tumor-initiating cell

## Abstract

Recent advances allowed culturing and examination of patient-derived colorectal cancer (PD-CRC) cells as organoids or spheroids. To be applied to practical personalized medicine, however, current methods still need to be strengthened for higher efficiency. Here we report an improved method to propagate PD-CRC tumor initiating cells (TICs) in spheroid culture. We established > 100 cancer spheroid lines derived from independent colorectal cancer patients employing a serum-containing medium with additional inhibitors, Y27632 and SB431542. Because colorectal cancer spheroids showed wide-range growth rates depending on the patient tumors, we searched for supplementary factors that accelerated proliferation of slow-growing CRC-TIC spheroids. To this end, we introduced a convenient growth-monitoring method using a luciferase reporter. We found that epidermal growth factor (EGF) and/or basic fibroblast growth factor (bFGF) were critical for steady propagation of a subset of CRC-TIC spheroids carrying the wild-type *RAS* and *RAF* genes. We also identified 5′-(*N*-ethyl-carboxamido)-adenosine (NECA), an adenosine receptor agonist, as an essential supplement for another subset of spheroids. Based on these results, we propose to optimize culture conditions for CRC-TIC spheroids by adjusting to the respective tumor samples. Our method provides a versatile tool that can be applied to personalized chemotherapy evaluation in prospective clinical studies.

## INTRODUCTION

Colorectal cancer is one of the commonest cancers worldwide [1]. Although the mortality of colorectal cancer is decreasing thanks to early detection efforts and treatment improvements, the disease is often lethal once it reaches the metastatic stage, even with molecular-targeted therapeutics such as antibodies against epidermal growth factor receptor (EGFR) and/or against vascular endothelial growth factor (VEGF) [2]. Furthermore, therapies remain to be developed that target the three commonly mutated gene products, APC, p53, and KRAS [3]. Recently, the “precision medicine” or “personalized approach” was introduced in order to improve the current colorectal cancer treatment. While the next generation sequencing technology has enabled us to identify the genetic changes in individual cancer cases [3], it is still challenging to predict therapeutic efficacies based on such information because of the diversity in genetic mutations and unknown epigenetic changes in each patient tumor [4, 5].

Alternatively, patient-derived xenografts (PDXs) have been employed to search for efficacious personalized therapies [6, 7]. Yet, it takes 1–4 months to establish the primary PDXs, and needs additional passages to expand them for the drug sensitivity tests [8]. It has become a practical alternative to culture patient-derived tumor initiating cells of the colorectal cancer (PD-CRC-TICs) as organoids or spheroids [9–12]. However, their clinical applications have been hampered by some technical difficulties, including the requirement for supplements such as growth factors, minerals, vitamins, and hormones. To propagate tissue-derived intestinal epithelial cells rapidly, a recent method employed a serum-containing medium conditioned by L-WRN fibroblasts that secrete three components (Wnt3a, R-spondin 3, and Noggin) essential for tissue stem cells [13]. Such media can support the proliferation of various types of epithelial stem cells from humans and mice when supplemented with a Rho-associated coiled-coil protein kinase (ROCK) inhibitor, Y27632, and a transforming growth factor β (TGF-β) type I receptor inhibitor, SB431542 [14].

In the present study, we have employed a serum-containing medium supplemented with the above two inhibitors (Y27632 and SB431542), and cultured PD-CRC-TICs as epithelial spheroids. We have established CRC-TIC spheroid lines that can be stored and utilized for further genetic and pharmacological analyses. We have also developed a convenient growth-monitoring method for human colonic spheroids, and identified novel supplementary factors that improved the proliferation of some slow-growing spheroid lines.

## RESULTS

### A simple medium helps propagate patient-derived colorectal cancer spheroids

**Figure 1:**
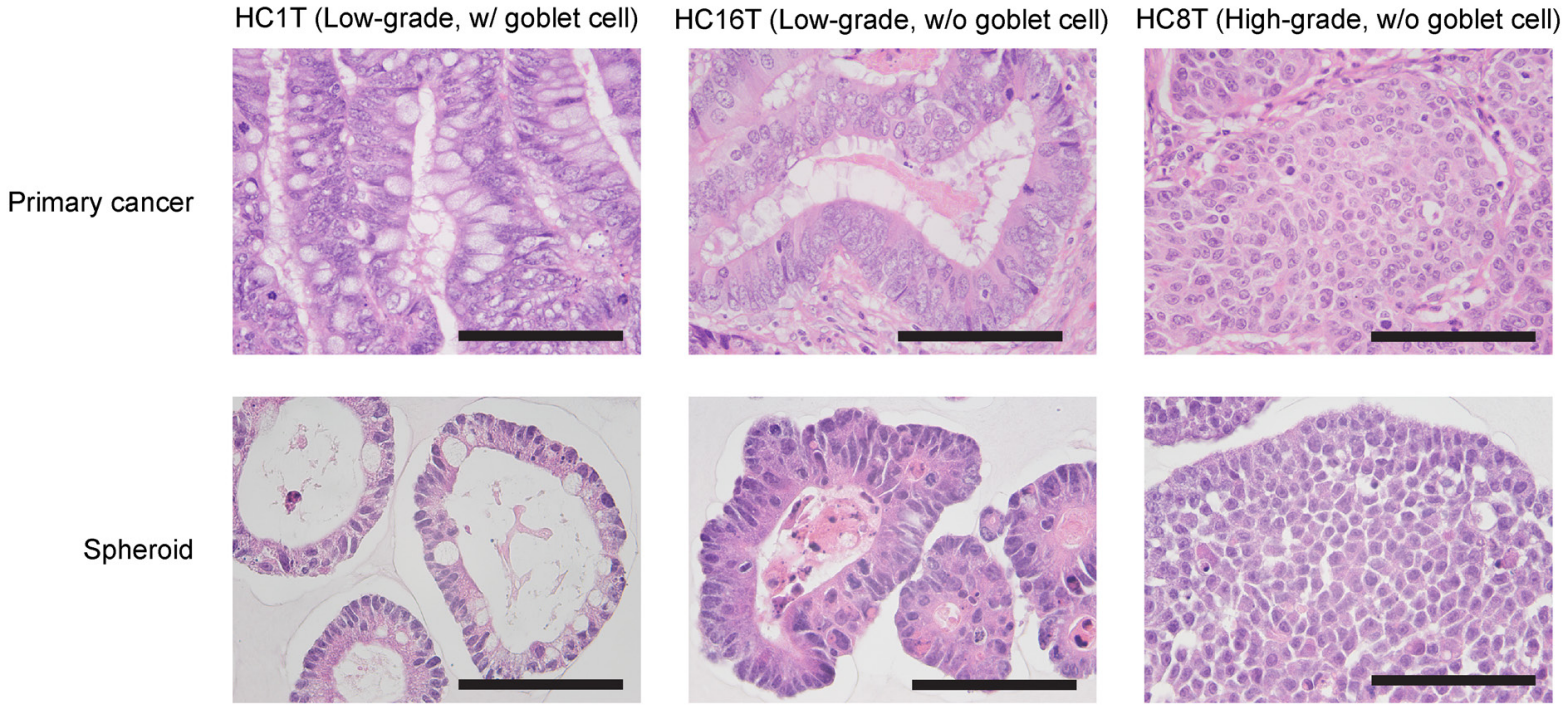
Histological characterization of patient-derived CRC-TIC spheroids Shown are pairs of H&E-stained specimens of the primary tumors (top) and spheroids (bottom) derived from the same patients. There were three representative histotypes; well-differentiated types, with (left) and without (center) mature goblet cells, and a poorly-differentiated type (right). Scale bar, 100 μm.

To culture patient-derived tumor initiating cell spheroids of the colorectal cancer (PD-CRC-TIC spheroids), we prepared “the cancer medium” in which the basal medium (Advanced DMEM/F12) was supplemented with FBS (5%) and two inhibitors, Y27632 and SB431542. For spheroids of the normal colonic epithelium from the same patients, we also prepared “the eL-WRN medium” (“e” for enhanced) by supplementing the L-WRN conditioned medium with above two inhibitors [15]. We attempted to culture 148 colorectal cancer samples from 141 patients. Pathologically, they were classified as low-grade (138/148), high-grade (5/148), or mucinous (5/148) adenocarcinomas according to the standard histological criteria [16, 17]. As anticipated, the histological features of the cancer epithelium coincided well between the primary tumors and their spheroids (Figure 1). To characterize the key genetic mutations, we sequenced the genomic regions of 36 CRC-TIC spheroid lines covering mutational hot spots of 50 cancer-related genes (Supplementary Figure 1). Our overall success rate for establishing colorectal cancer spheroids was 73% (108/148), whereas that for normal epithelial stem cells was 93% (124/134). Notably, our success rate for 24 cases in the latest quarter reached 88% (Table 1; see below and Discussion). The reasons for unsuccessful CRC-TIC spheroid culture included contamination by microorganisms (bacteria or yeasts; 4 cases), apparent cell damage by radiation therapy before surgery (2 cases), and poor cell growth followed by cell death in early passages (of unknown causes; 34 cases) (see Discussion). We found no statistical correlations between the success rates and tumor stages (Table 2).

**Table 1:** Success rates for establishing CRC-TIC spheroid culture scored by period

Period	11/13/2014– 6/30/2015	7/1/2015– 12/31/2015	1/1/2016– 6/30/2016	7/1/2016– 12/31/2016	1/1/2017– 3/31/2017
Total attempts	22	45	34	23	24
^*^Success (%)	14 (64%)	31 (69%)	26 (76%)	16 (70%)	21 (88%)

^*^ Numbers of colorectal cancer samples from which spheroid lines were successfully established.

**Table 2:** Tumor stages of primary colorectal cancer samples

Stage	I	II	III	IV	Total
Number	17	64	48	19	148
^*^Success (%)	15 (88%)	42 (66%)	37 (77%)	14 (74%)	108 (73%)

^*^ Numbers of colorectal cancer samples from which spheroid lines were successfully established.

### Introduction of luciferase enables monitoring of precise spheroid growth rates

**Figure 2:**
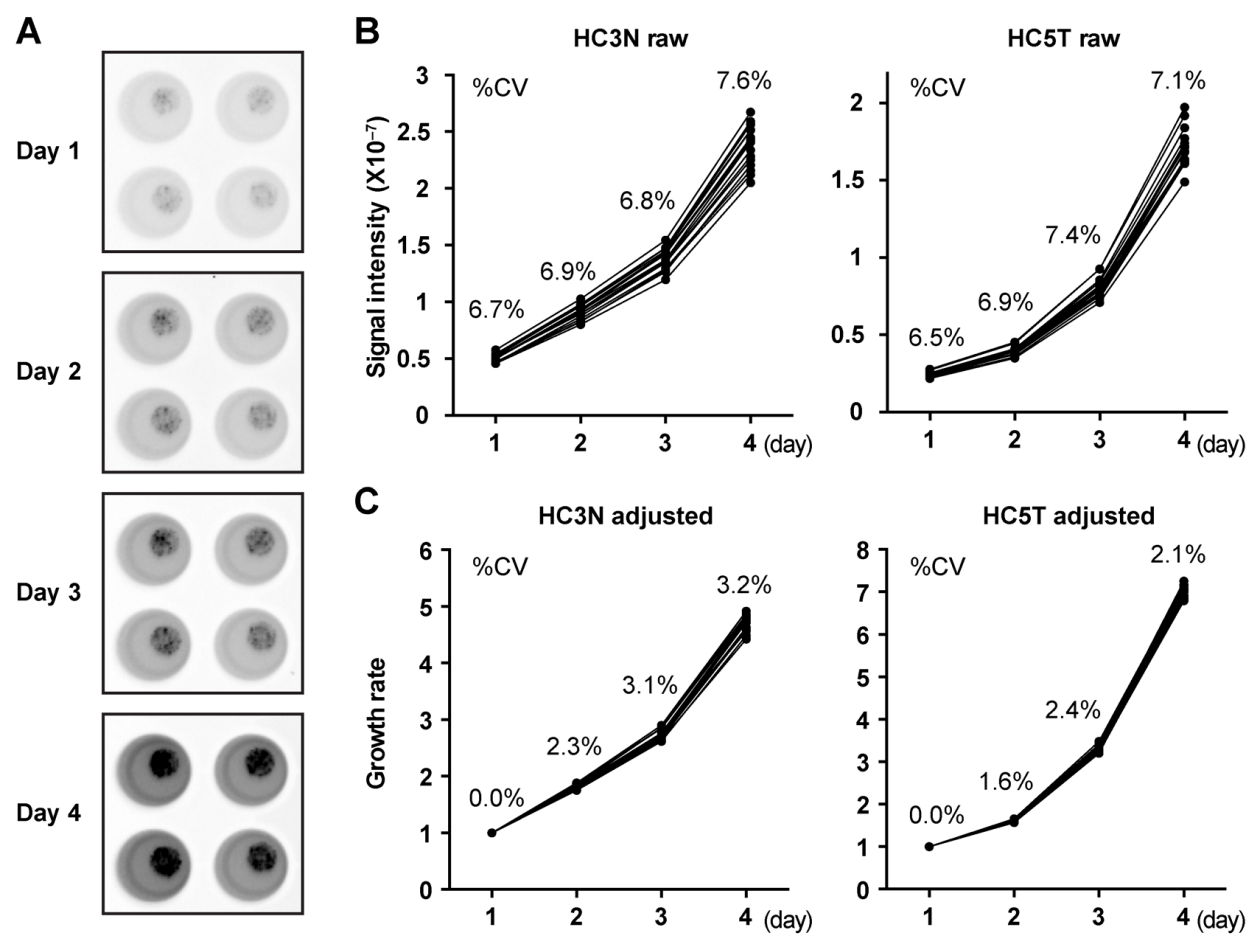
An accurate growth-monitoring method for human spheroids **(A)** Quantitative presentation of bioluminescence from luciferase-expressing spheroids from days 1 to 4 after passages into the wells of a 96-well cell culture plate. The gray level in each well represents direct photon counts for the spheroids embedded in the corresponding Matrigel drop. The total luminescence represents the cell number in each well. (**B, C**) Growth monitoring of luciferase-expressing spheroids. Photon counts from spheroid lines derived from normal (HC3N raw; left) and cancer (HC5T raw; right) SCs were scored daily during post-passage days 1–4 (B). Graphs in (C) show growth rates calibrated to the initial cell numbers based on the photon counts on day 1. Sixteen replicated wells were tested for each line. The coefficient of variation (%CV) is shown for each data point.

We noticed that individual cancer spheroid lines varied widely in growth rate *in vitro*. However, it was difficult to determine cell number in 3D culture. It was also technically challenging to distribute the spheroid cells equally to multiple culture wells because trypsinized spheroids contained cell aggregates of various sizes, causing wide statistical errors in quantifications such as MTT assays. To minimize such errors, we employed a method which canceled out the variations in the initial cell numbers. Practically, we utilized spheroids infected with a lentivirus containing a luciferase expression vector driven by the CAG promoter [18]. Because its high efficiency of infection eliminated the necessity to enrich expressor subpopulations [15], we passaged post-infection spheroids 2–3 times without subcloning, and directly subjected them to cell growth assays. We monitored the growth of the normal and cancer spheroid lines daily from post-passage days 1 to 4 (Figure 2A). As shown in Figure 2B, bioluminescence levels among 16 replicated wells varied rather widely, reflecting differences in the initial cell numbers (coefficient of variation, 6.5–7.6%). However, when calibrated to the initial photon counts on day 1, the variations were reduced to 1.6–3.2% (Figure 2C). Thus, the present method provides a highly reproducible alternative to cell-disruptive methods. Accordingly, we have established a reliable method to monitor human CRC-TIC spheroid growth which can be performed with a small number of replicates.

### EGF and bFGF can stimulate the growth of CRC-TIC spheroid subpopulations

**Figure 3:**
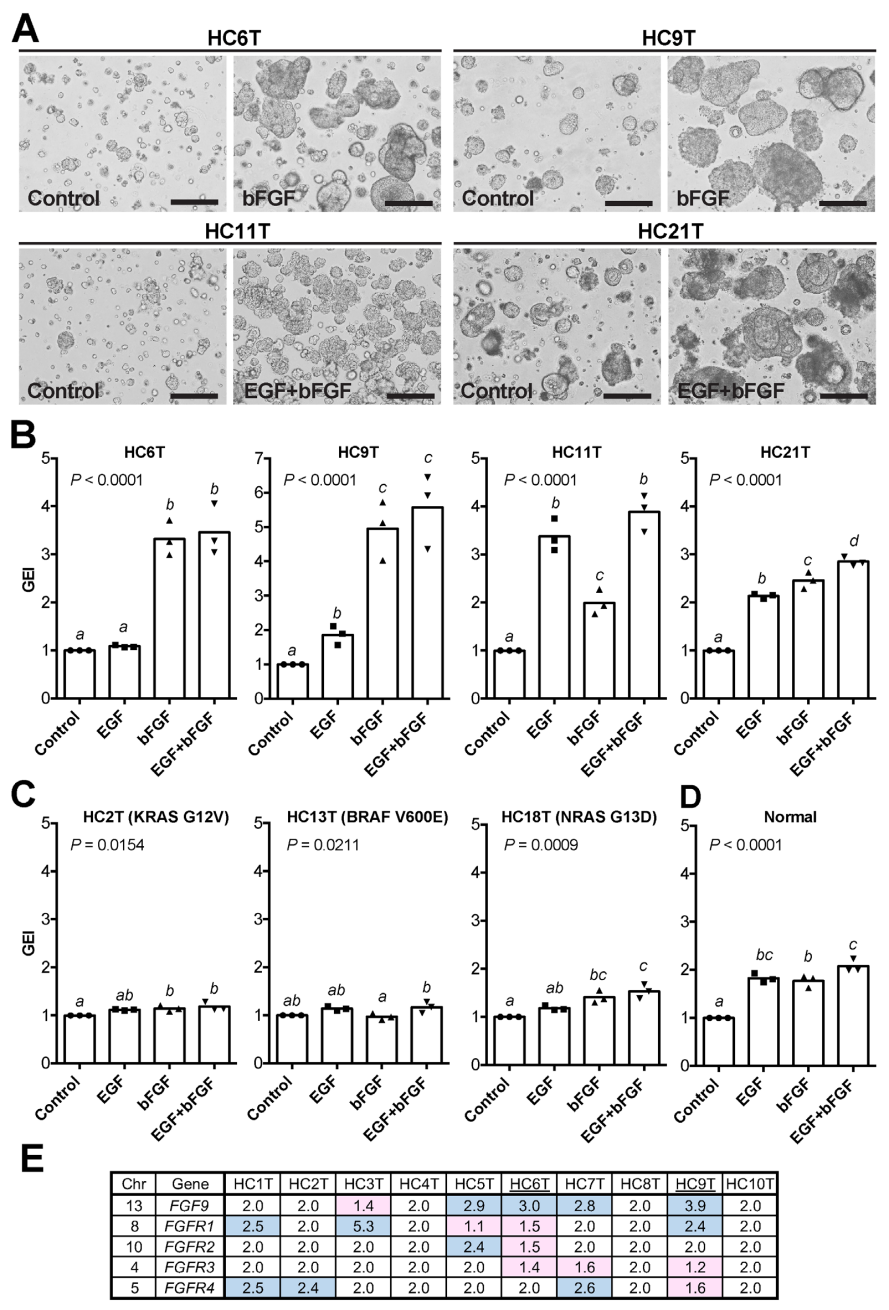
Growth-promoting effects of EGF and bFGF on CRC-TIC spheroids **(A)** Representative phase-contrast micrographs of four cancer spheroid lines cultured in the presence of EGF and/or bFGF for 8 days. Scale bar, 200 μm. **(B)** Growth effect indices (GEI) of CRC-TIC spheroid lines carrying the wild-type *RAS*/*RAF* genes. **(C)** GEI of the spheroid lines carrying mutant *RAS*/*RAF* genes. **(D)** GEI of normal colonic epithelial SC spheroids. Plotted are the GEI with means in three independent experiments (B and C) or spheroid lines (D). Data were analyzed using one-way ANOVA (*P* values are shown in the graphs) followed by Tukey’s post-test. The statistical significance between the mean values are indicated by *a*, *b*, *c*, and *d*; the value differences between different letters are statistically significant (*P* < 0.05), whereas those between the same are not. **(E)** Copy numbers of the *FGF9*, *FGFR1*, *FGFR2*, *FGFR3*, and *FGFR4* gene loci in ten CRC-TIC-spheroid lines. Gain (blue) or loss (red) of the chromosomal region was determined relative to the control genomic DNA.

EGF and bFGF have been frequently added to non-serum culture media to support the cell growth of various types [19]. However, it was unclear whether they helped spheroid proliferation in our serum-containing cancer medium. To investigate this issue, we cultured with EGF and/or bFGF four slow-growing CRC-TIC spheroid lines that carried wild-type *RAS*/*RAF* genes. Because EGF/bFGF facilitated the growths of these spheroid lines substantially (Figure 3A), we then quantified their effects by the luminescence-based growth monitoring. To calibrate the effects, we introduced the growth effect index (GEI) defined as the relative growth rate of a treated group to that of its solvent-only control in each paired assay. We found that additions of both EGF and bFGF more than doubled the GEI in all four spheroid lines carrying wild-type *RAS*/*RAF* (Figure 3B and Supplementary Figure 2A). Notably, the extents of growth promotion by EGF and/or bFGF were different depending on the spheroid line carrying wild-type *RAS*/*RAF*. In two lines (HC6T and HC9T), bFGF was more effective than EGF, whereas in another (HC11T), vice versa (Figure 3B). In another spheroid line (HC21T), the effects were similar between the two growth factors (Figure 3B). As anticipated, EGF/bFGF showed only limited growth-promoting effects on three spheroid lines that carried *RAS* or *RAF* mutations (< 55% increases by both; Figure 3C and Supplementary Figure 2B). Interestingly, EGF and bFGF doubled the growth of normal epithelial stem cell (SC) spheroids, too (Figure 3D and Supplementary Figure 1C). Consistent with a previous report [20], four of ten spheroid lines showed gain of the *FGF9* chromosomal region (Figure 3E), which may explain better GEI by bFGF at least in two spheroid lines (HC6T and HC9T) compared with those in normal spheroids. However, copy number alterations of FGF receptor genes were not correlated with bFGF sensitivity (Figure 3E).

### Cell autonomous activity of the canonical Wnt signaling does not necessarily correlate with mitotic activity in CRC-TIC spheroid lines

**Figure 4:**
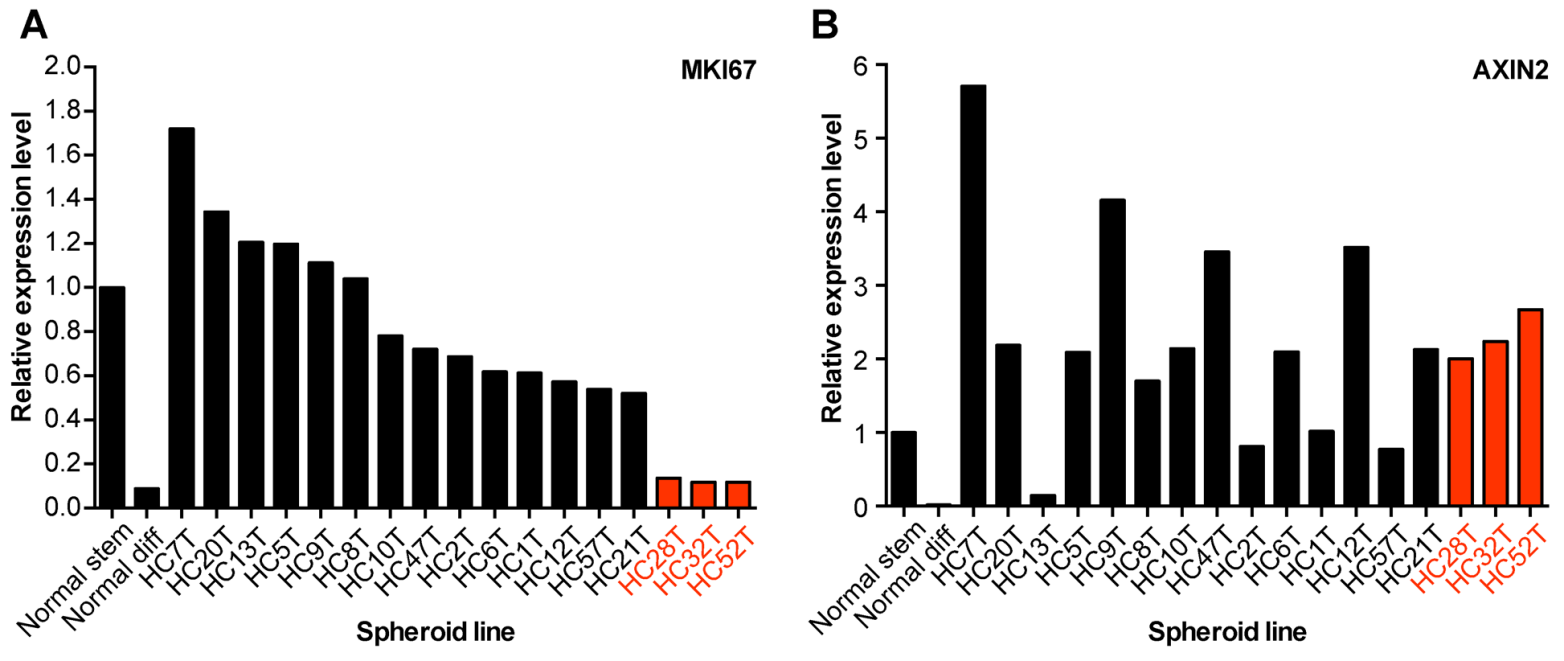
Ligand-independent Wnt-signaling activation in CRC-TIC spheroid lines **(A, B)** Expression levels of *MKI67* (Ki-67) (A) and *AXIN2* (B) mRNAs. Normal colonic epithelial SC spheroids were cultured in the eL-WRN medium (Normal stem) or the differentiation medium (Normal diff), whereas CRC-TIC spheroids were cultured in the cancer medium supplemented with EGF and bFGF. Shown are the mean expression levels in triplicates relative to those in normal colonic epithelial SCs (Normal stem). Data of three slow-growing CRC-TIC lines are presented in red.

The canonical Wnt signaling plays crucial roles in the proliferation of CRC-TICs although they require no exogenous Wnt ligands due to mutations in the *APC* or β-catenin gene [21]. The strength of cell-autonomous growth signals can vary depending on the mutations or epigenetic changes in individual colorectal cancer cases, which may explain why some spheroid lines were difficult to be maintained even in the presence of EGF and bFGF. Thus, we hypothesized that the canonical Wnt signaling activity was weak in such spheroid lines, and determined the levels of *MKI67* (Ki-67) and *AXIN2* mRNA in our spheroid lines by qRT-PCR analyses, because *AXIN2* is an exclusive target of the canonical Wnt signaling pathway [13, 15, 22, 23]. Surprisingly, three slow-growing spheroid lines with low *MKI67* levels expressed even higher levels of *AXIN2* mRNA than the normal colonic SCs (Figure 4A, 4B), excluding the possibility that their growth was compromised by weak canonical Wnt signaling.

### Chemical library screening identified candidates for growth-promoting supplements

**Figure 5:**
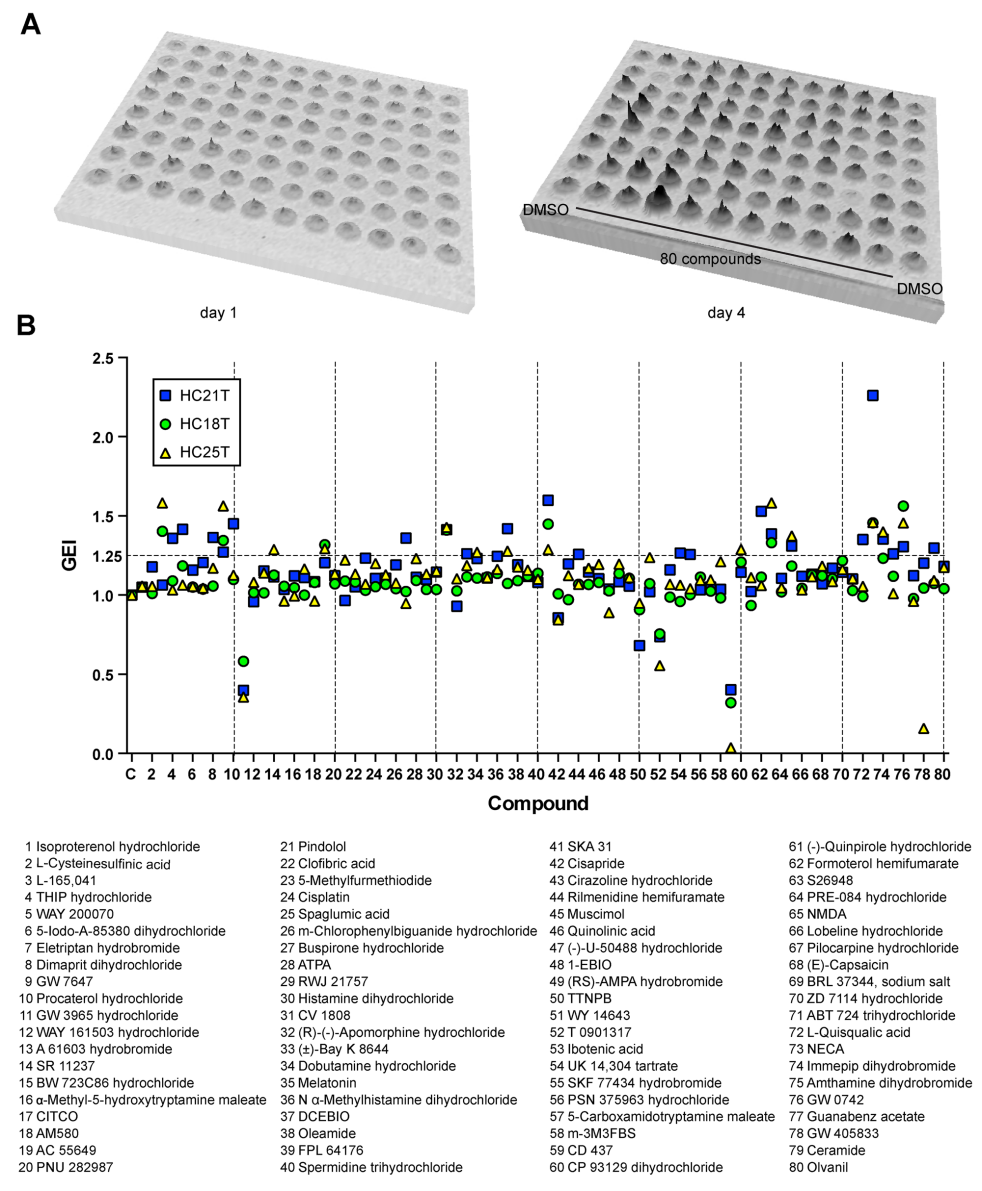
Screening for growth-stimulating compounds on CRC-TIC spheroids **(A)** Computerized presentation of bioluminescence from a luciferase-expressing CRC-TIC spheroid line (HC21T) upon incubation with the test compounds separately at 10 μM each. Bioluminescence was detected before (day 1, left) and after (day 4, right) the drug treatment. The summit in each well represents direct photon counts for the spheroids embedded in each Matrigel drop. **(B)** Effects of eighty compounds on the growths of three CRC-TIC spheroid lines. Shown are the GEI in three independent spheroid lines (top) and the list of compounds (bottom).

**Figure 6:**
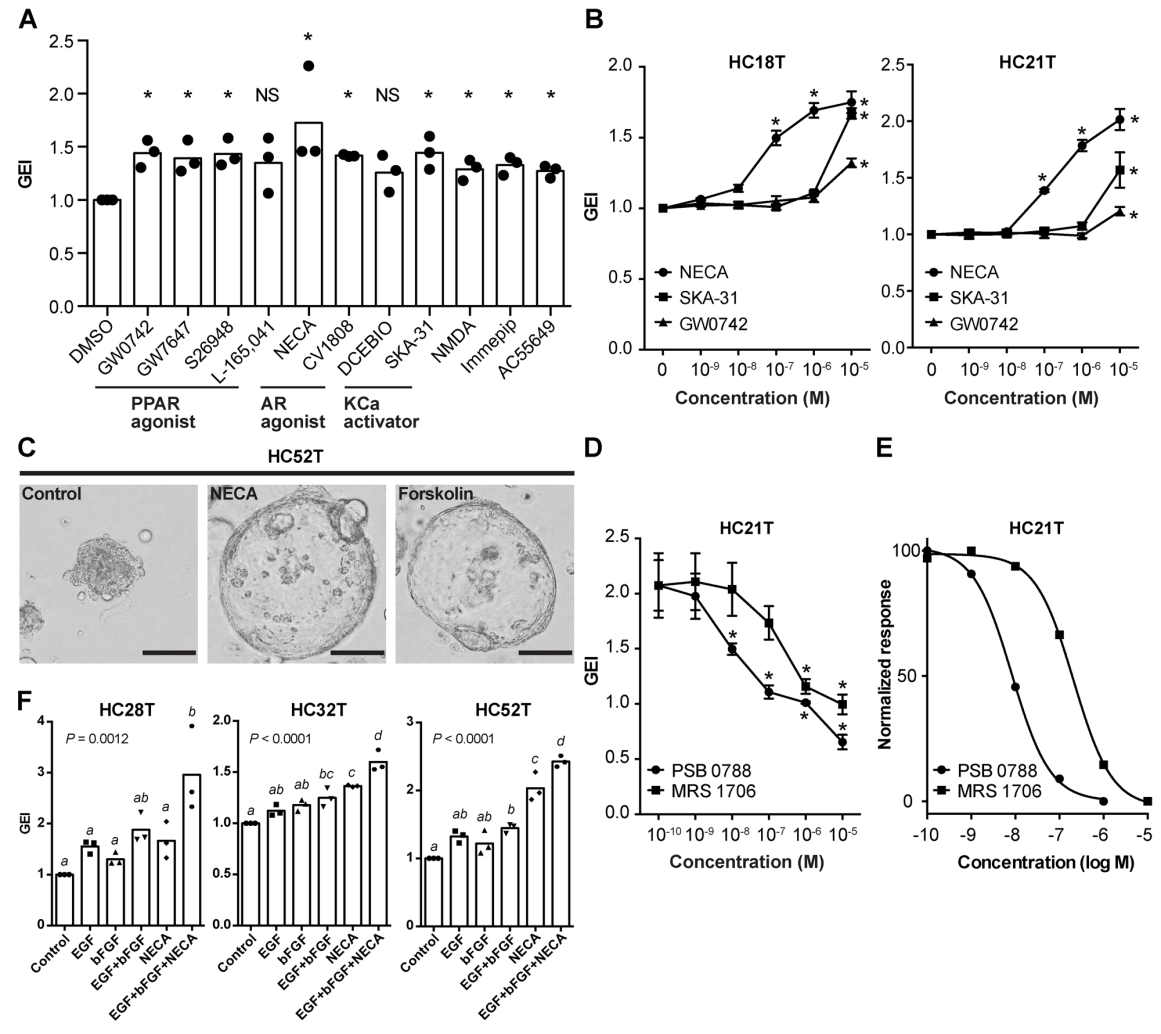
Evaluation of chemical compounds that facilitate the growth of CRC-TIC spheroids **(A)** Growth-promoting effects of the compounds identified in Figure 5B. Shown are the data sets with mean values > 1.25 (i.e., > 25% increase in growth). More than one compounds in each of three groups showed growth stimulation: peroxisome proliferator-activated receptor agonist (PPAR agonist); adenosine receptor agonist (AR agonist); calcium-activated potassium channel activator (KCa activator). Plotted are the GEI with means in three independent spheroid lines. Asterisks indicate statistically significant differences from the solvent-only (0 μM) spheroids (*P* < 0.05; analyzed using Student’s *t*-test). ND, no significant difference. **(B)** Dose-dependent effects of the selected three compounds on the growths of two colorectal cancer spheroid lines (HC18T and HC21T). Shown are the mean GEI ± SD in three independent experiments. Asterisks indicate statistically significant differences from the solvent-only (0 μM) spheroids (*P* < 0.05; analyzed using one-way ANOVA followed by Tukey’s post-test). **(C)** Representative phase-contrast micrographs of spheroids after NECA or forskolin treatment. Colorectal cancer spheroids (HC52T) were cultured with solvent only, 1 μM NECA, or 1 μM forskolin for five days. Note the increased sizes of NECA- and forskolin-treated spheroids caused by cell flattening. Scale bar, 100 μm. **(D, E)** Dose-dependent inhibitory effects of adenosine receptor antagonists (PSC 0788 and MRS 1706) on the growth of CRC-TIC spheroids (HC21T) cultured in the presence of 1 μM NECA. Shown are the mean GEI ± SD (D) and fitted curves of the normalized mean values (E) in three independent experiments. Asterisks in (D) indicate statistically significant differences from the solvent-only (0 μM) spheroids (*P* < 0.05; analyzed using one-way ANOVA followed by Tukey’s post-test). **(F)** Growth stimulation by three supplementary factors on slow-growing spheroid lines. Luciferase-expressing spheroids were cultured in the presence of the indicated factor(s) for 3 days. Plotted are the GEI with means in three independent experiments calculated from the same data sets in Supplementary Figure 3E. Data were analyzed using one-way ANOVA (*P* values are shown in the graphs) followed by Tukey’s post-test. The mean values between the different letters are statistically different (*P* < 0.05).

To find additional supplementary factors that could promote spheroid growths, we screened 80 pharmacologic agonists/activators for growth-promoting effects on three luciferase-expressing CRC-TIC spheroid lines with moderate growth rates (i.e., 3–4 times in 3 days). We identified eleven compounds that stimulated spheroid growths by > 25% (Figure 5A, 5B). These included four peroxisome proliferator-activated receptor (PPAR) agonists, two adenosine receptor (AR) agonists, and two calcium-activated potassium-channel (KCa) activators (Figure 6A). We then performed titration experiments for GW0742 (PPARδ agonist), 5′-(*N*-ethyl-carboxamido)-adenosine (NECA, non-selective AR agonist), and SKA-31 (activator of KCa2 and KCa3.1a). While NECA exhibited a strong activity of growth promotion (EC_50_ = 63.1 and 195 nM with HC18T and HC21T spheroid lines, respectively), other two compounds showed significant effects only at the maximum dose of 10 μM (Figure 6B). We also confirmed that another AR agonist, CV1808 (2-phenylaminoadenosine), showed a growth-promoting effect though weaker than that of NECA (Supplementary Figure 3A). These results suggest that NECA is a candidate suitable for CRC-TIC spheroid cultures. Notably, dose-dependent responses for NECA in three independent experiments were highly reproducible (Supplementary Figure 3B), underscoring the reliability of our spheroid culture in drug-sensitivity tests.

### NECA supports CRC-TIC spheroid growth through cAMP-PKA signaling pathway

Because A_2B_ is the only adenosine receptor expressed in the colonic epithelium, NECA appears to stimulate the cyclic AMP (cAMP)-protein kinase A (PKA) signaling in these cells [24]. Consistently, NECA as well as CV1808 induced morphological changes in spheroids characterized by cell flattening and increased sphere size, similar to those in forskolin-treated spheroids (Figure 6C and Supplementary Figure 3C) [25]. The growth-promoting effect of NECA was specifically antagonized by two A_2B_-specific inhibitors, PSB 0788 and MRS 1706 (IC_50_ = 8.32 and 210 nM with HC21T spheroid line, respectively; Figure 6D, 6E, and Supplementary Figure 3D).

To evaluate NECA further, we applied the NECA-containing medium to three slow-growing spheroid lines with which EGF and bFGF alone were ineffective in growth stimulation (Figure 4A, red bars). Supplementation with NECA in addition to EGF and bFGF enabled us to maintain these spheroid lines stably. Accordingly, we were able to infect them with a lentivirus, and make them express the luciferase gene. By luciferase assays, we confirmed that addition of three supplementary factors stimulated their growths by > 50% (Figure 6F). The increments of GEI were critical for their propagation because they barely grew in the cancer medium alone (growth rates, ∼1.0) (Supplementary Figure 3E). These results suggest that activation of the cAMP-PKA and mitogen-activated protein kinases (MAPK)/extracellular signal-regulated kinases (ERK) signaling enhances proliferation of CRC-TIC spheroids in an additive manner though not synergistic. Thus, we can cultivate even some extremely slow-growing spheroids by supplementing the basal cancer medium with three additional ingredients, EGF, bFGF, and NECA.

### Optimized culture conditions for the patient-derived spheroids

**Figure 7:**
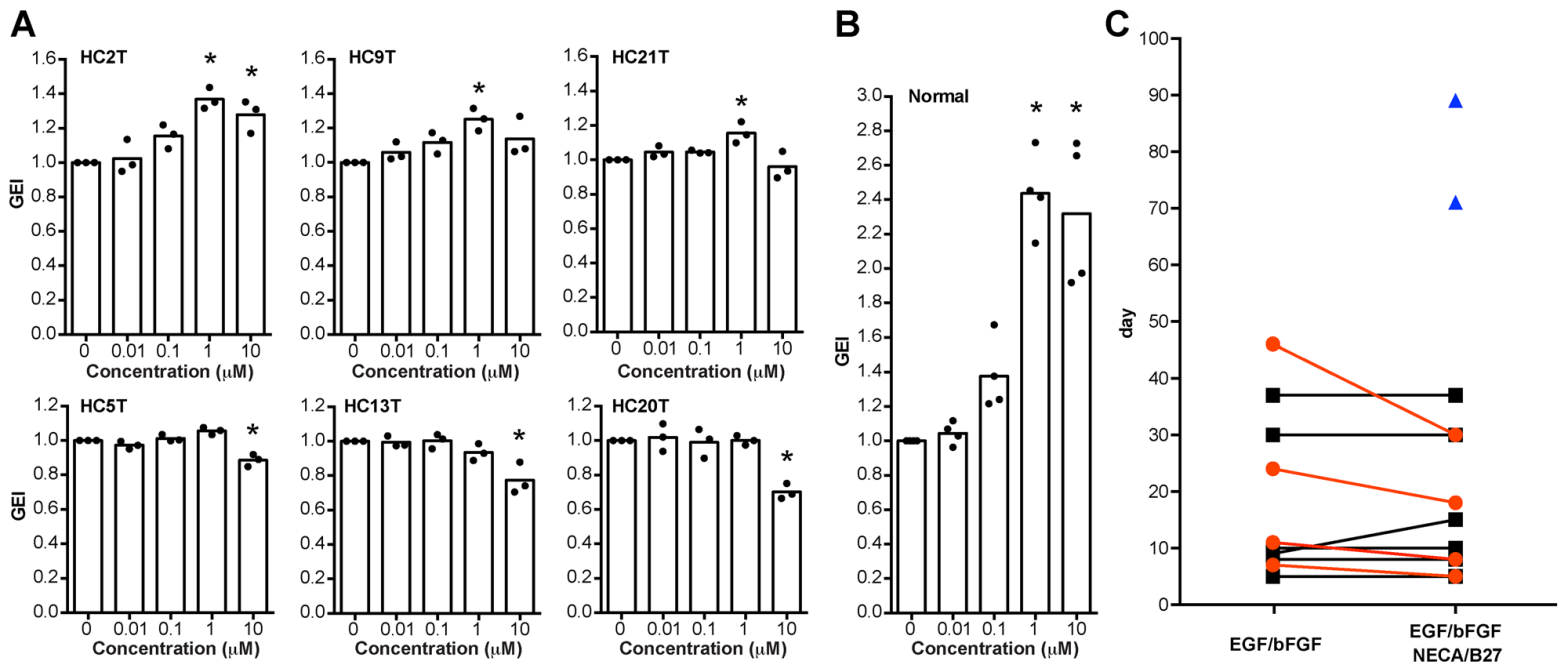
Optimization of spheroid culture conditions **(A, B)** Dose-dependent effects of SB431542 on the growths of cancer (A) and normal (B) spheroid lines. Luciferase-expressing cancer and normal spheroids were cultured in the EGF/bFGF-containing cancer medium and the eL-WRN medium, respectively, in the presence of SB431542 at the indicated concentrations. Plotted are the GEI with means in three independent experiments (A) or those in four independent spheroid lines (B). Asterisks indicate statistically significant differences from the solvent-only (0 μM) spheroids (*P* < 0.05; analyzed using one-way ANOVA followed by Tukey’s post-test). Note that the effects on cancer spheroids are less than those on normal spheroids. **(C)** Growth-supportive effects of NECA and the B27 supplement for culture initiation of colorectal cancer tissues. Epithelial cells separated from colorectal cancer tissues were cultured with the EGF/bFGF-containing cancer medium in presence or absence of NECA and the B27 supplement. Plotted are times needed for expansion of spheroids to four wells of a 12-well cell-culture plate. Of eleven cases tested, supplementation of NECA/B27 was essential to establish spheroid lines in two cases (blue triangles), promoted faster spheroid growth in four cases (red circles), and showed no supportive effect in other five cases (black squares).

**Figure 8:**
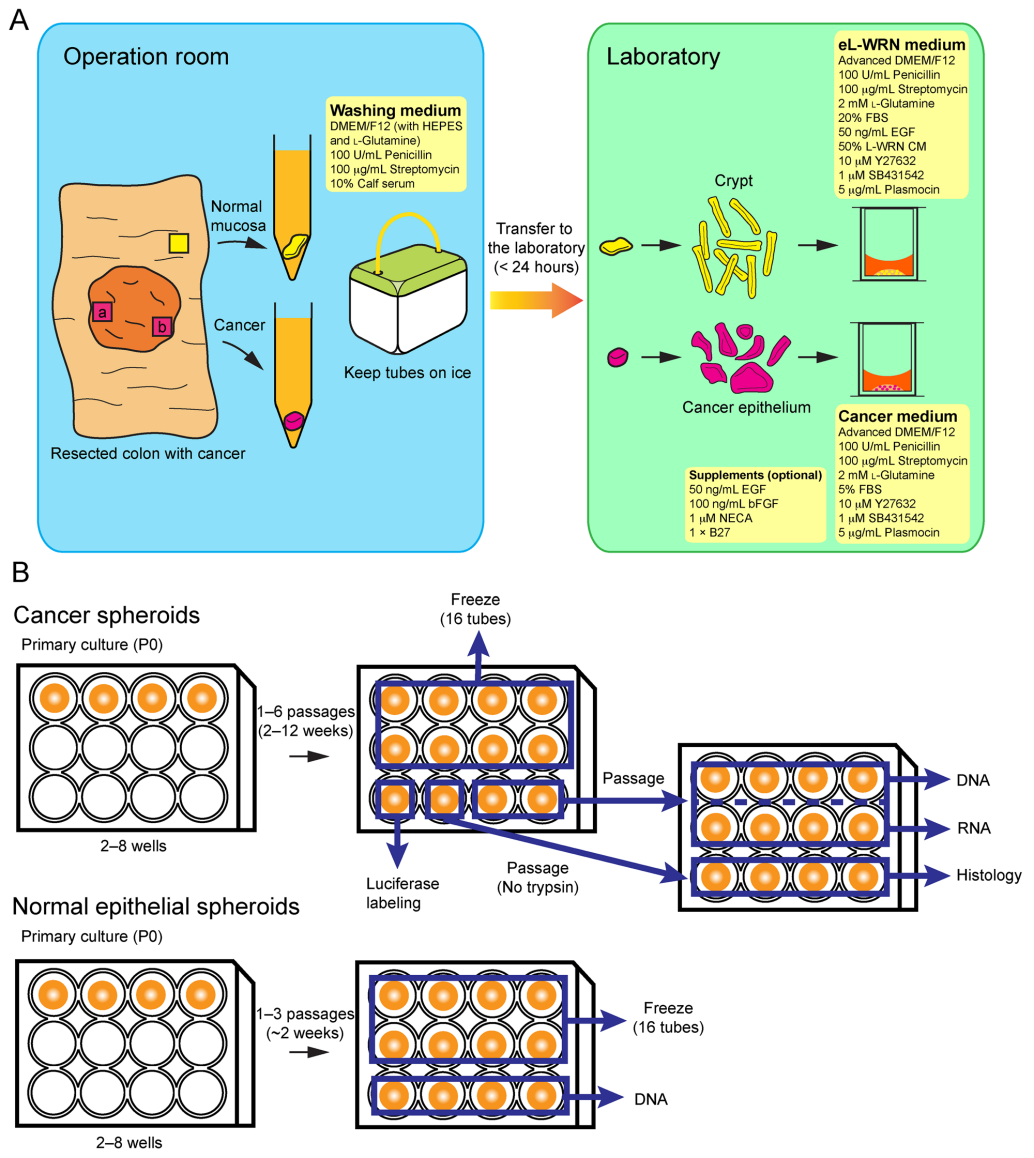
A schematic workflow for establishing patient-derived CRC-TIC spheroids **(A)** The outline of procedures in the operation room and laboratory. We recommend collecting tumor samples from at least two different tumor regions (a and b). **(B)** Details for the spheroid propagation and sample preparation processes. For preparation of histological specimens, spheroids should be passaged without trypsinization so that large aggregates are formed (see Figure 1).

On the basis of the above results, we modified our previous culture media for both normal and cancer SC spheroids. A ROCK inhibitor, Y27632, is required to maintain human colorectal cancer stem cells [26, 27], as well as human gastrointestinal stem cells [15]. We found that Y27632 also increased the growths of CRC spheroids (6 of 12 lines tested) at a dose of 10 μM (Supplementary Figure 4A). A TGF-β type I receptor inhibitor, SB431542, is essential for long-term culture of human normal gastrointestinal spheroids [15]. On the other hand, SB431542 supported the growths of some CRC spheroid lines (3 of 10 lines tested) although its effects were not as dramatic as that on normal colonic spheroids (Figure 7A, 7B, and Supplementary Figure 4B). We optimized its concentration to 1 μM to avoid its growth inhibition often observed at 10 μM (Figure 7A and Supplementary Figure 4B). To the eL-WRN medium for the normal epithelial SC spheroids, we added EGF because it often promoted their growth, too (Figure 3D). In addition, NECA and the B27 supplement, a cocktail of antioxidants, supported establishment of spheroid lines that grew poorly in early passages (Figure 7C). Therefore, we recommend including EGF, bFGF, NECA, and the B27 supplement in the initial culture of PD-CRC-TICs, and determining later whether these growth factors are essential to maintain them. Owing to these optimizations, our success rate for establishing CRC-TIC spheroids increased to ∼90% during the latest quarter (Table 1). A schematic workflow is summarized in Figure 8A and 8B for culturing CRC-TIC spheroids and normal colonic epithelial SC spheroids.

## DISCUSSION

In the present study, we developed an improved method for establishing human CRC-TIC spheroids, using a simple culture medium with minimal supplementary factors. Namely, our basal cancer medium consisted of “Advanced DMEM/F12” supplemented with Y27632, SB431542, and FBS. The ROCK inhibitor, Y27632, was reported to inhibit anoikis of dissociated human embryonic stem cells [28], and has been used for culturing normal human intestinal organoids/spheroids [15, 29]. The TGF-β type I receptor inhibitor, SB431542, appears to help CRC-cell proliferation that can be inhibited by TGF-β signaling [30, 31]. Supplementation with these two inhibitors supported the growth of mouse intestinal adenoma spheroids in the same serum-containing medium [14, 32], hence the present application to human CRC-TIC cells as well.

Although the cancer medium enabled us to culture > 70% of the colorectal cancer samples into TIC spheroids, not all of them were successful. Poor spheroid growth in early passages was the commonest cause of unsuccessful culture. One of the conceivable reasons is that the resected tissue samples contained few TICs. Such tissue pieces may be composed mostly of normal epithelial cells, stromal cells, or differentiated cancer cells such as mature goblet cells in case of low-grade adenocarcinoma (Figure 1). To avoid this problem, we recommend collecting more than one tissue samples from different regions of a tumor (Figure 8A). Another conceivable reason is lack of the critical factor(s) that supports adaptation of colorectal cancer cells to the *in vitro* culture microenvironment. To address this issue, we searched for additional supplementary factors that increased the efficiency in establishing CRC-TIC spheroid lines. To this end, we developed an accurate but simple growth-monitoring method for 3D-cultured spheroids; employment of GEI based on luciferase bioluminescence.

Two signaling pathways play central roles in intestinal epithelial cell growth *in vitro*; namely, those of the canonical Wnt signaling and MAPK/ERK [33]. Although a recent study reported that a subpopulation of colorectal cancer organoids was dependent on Wnt ligands for their growth [11], our attempts to culture CRC-TIC spheroids in the Wnt-containing L-WRN medium resulted in predominant propagation of the normal epithelial cells that remained in the tumor tissue. On the other hand, we found that EGF and bFGF effectively enhanced the growth of CRC-TIC spheroids carrying wild-type *RAS*/*RAF* genes. Interestingly, we identified multiple colorectal cancer spheroid lines that depended on bFGF for their growth rather than on EGF (Figure 3B), suggesting that FGFR inhibitors may suppress the growth of CRC-TICs, through blocking the Ras/Raf/ERK pathway [34]. Consistently, a recent study showed that *FGF9* gene amplification was associated with resistance of colorectal cancer cells to cetuximab [20]. Notably, at least two of our spheroid lines (HC6T and HC9T) that responded well to bFGF contained such gains of its chromosomal region (Figure 3E). Thus, the combination of an FGFR inhibitor and cetuximab can be more efficacious in treating colorectal cancer with wild-type *RAS*/*RAF* genes. Our method should help screen patients suitable for such a therapy.

In addition, we found that NECA, an adenosine receptor agonist, accelerated growth of some slow-growing spheroids (Figure 6D). The characteristic changes in the spheroid morphology by adenosine receptor agonists strongly suggest involvement of the cAMP-PKA pathway [25]. Activation of cAMP-PKA signaling induces metabolic changes that confer resistance to glucose deprivation and anoikis on cancer cells [35, 36]. Consistently, we noticed that supplementation with NECA was effective in case spheroid cells grew poorly in early passages (Figure 7C), suggesting the role of NECA in CRC-TIC adaptation to *in vitro* culture. Because A_2B_ antagonists canceled the growth-promoting effect of NECA (Figure 6D, 6E), and can inhibit the growth of prostate cancer and colonic adenocarcinoma cells [37, 38], the adenosine receptor inhibitor may be one of promising candidates in cancer therapy.

In conclusion, we developed an efficient and low-cost propagation method for CRC-TIC spheroids. With the optimized culture protocols, our success rate for establishing colorectal cancer spheroids has increased to ∼90% (Table 1). Accordingly, it has become a practical and feasible strategy to exploit PD-CRC-TICs for clinical applications. Cultured spheroids consist of the pure cancer cell population without stromal cells, which provides an excellent source for high-quality DNA samples that enable unambiguous genetic diagnosis. Accurate mutational profiles of the primary CRC-TICs should help determine the chemotherapeutics more efficiently. When treatments with the current regimen fail, patient-derived “spheroid” xenografts (PDSX) may provide sensitivity tests for other available or novel therapeutic regimens. Compared with transplantation of the whole patient tumor tissues, that of CRC-TIC spheroids should provide test mice more suitable for precise evaluation of drug sensitivity personalized for each patient.

## MATERIALS AND METHODS

### Reagents and antibodies

The following reagents were purchased from commercial sources: luciferin (Promega, Madison, WI, USA), a chemical collection for agonists/activators (S990043-AGO1), 5′-(*N*-ethyl-carboxamido)-adenosine (NECA), 2-phenylaminoadenosine (CV1808), SKA-31 (Sigma, St. Louis, MO), Y27632, SB431542, GW0742, PSB 0788, MRS 1706 (R&D Systems, Minneapolis, MN). The L-WRN cells [13] were obtained from Dr. Thaddeus S. Stappenbeck (Washington University), and their conditioned medium (L-WRN CM) was prepared according to a reported protocol [14].

### Human samples

Tumor samples were obtained from a total of 141 colorectal cancer patients who underwent primary resections at Kyoto University Hospital (KUHP) between October 2014 and March 2017. Their diagnosis was confirmed as colorectal cancer through histopathologic examinations by board-certified pathologists at KUHP. The study protocols were approved by the institutional review board of KUHP, and patients provided written consents for investigational analyses.

### Initial spheroid culture of human CRC-TICs and normal colonic epithelial cells

From each surgical specimen that contained the colorectal cancer lesion(s) and surrounding tissues, ∼2 pieces of the tumor (0.5–4 cm^3^ each) and one piece of normal mucosa (∼4 cm^2^) were isolated, and transferred to the laboratory in the ice-cold washing medium [DMEM/F12 with HEPES and L-glutamine (Nacalai Tesque, Kyoto, Japan), 100 units/ml penicillin (Nacalai), 0.1 mg/ml streptomycin (Nacalai), and 10% calf serum (Sigma)]. Tissues were processed within 24 h after surgery. A piece of the colorectal cancer specimen (0.5–1.0 cm^3^) or a flap of the normal mucosa (1–2 cm^2^) was separated from the connective tissue, washed with PBS, and minced using scissors in a 60-mm petri dish. The tissue fragments were digested in 2 ml of collagenase solution [the washing medium supplemented with 0.2% collagenase type I (Thermo Fisher Scientific, Waltham, MA) and 50 μg/ml gentamicin (Thermo Fisher)] at 37 °C for 40–60 min, and dissociated by pipetting with a 1 ml-pipette with ∼20 min intervals 2–3 times. Then, epithelial cell clusters were filtered through a 100-μm cell strainer (Corning Inc., Corning, NY), and collected according to a previous report [15]. Epithelial cells were then resuspended in Matrigel (Corning), and placed in the center of each well of the 12-well cell-culture plate (30 μl per well; TPP, Trasadingen, Switzerland). After polymerization of Matrigel at 37 °C, epithelial cells were cultured with the cancer medium [Advanced DMEM/F-12 (Thermo Fisher), 100 units/ml penicillin, 0.1 mg/ml streptomycin, 2 mM L-glutamine, 10 μM Y27632, 10 or 1 μM SB431542, 5 μg/ml Plasmocin (Invivogen, San Diego, CA), and 5% FBS (Thermo Fisher)] or in the eL-WRN medium [Advanced DMEM/F-12, 100 units/ml penicillin, 0.1 mg/ml streptomycin, 2 mM L-glutamine, 50% L-WRN CM, 10 μM Y27632, 10 or 1 μM SB431542, 50 ng/ml EGF (Peprotech, Rocky Hill, NJ), 5 μg/ml Plasmocin, and 20% FBS]. When necessary, the cancer medium was supplemented with 100 ng/ml basic bFGF (Peprotech), 50 ng/ml EGF, 1 μM NECA, and/or 1 × B27 supplement (Thrmo Fisher) to support the growth of slow-growing spheroids. The differentiation medium for normal colonic epithelial spheroids were prepared as described previously [25]. The medium was changed every other day. Phase-contrast images of spheroids were captured through OLYMPUS IX70 microscope (OLYMPUS, Tokyo, Japan) equipped with OLYMPUS DP70 digital camera and DP Controller 1.2 software (OLYMPUS).

### Lentivirus infection of spheroids

A cDNA fragment encoding a firefly luciferase (*luc2,* Promega) was inserted into the pCX vector that contained a CAG promoter [18] to construct pCX-Luc2. The expression unit (CAG-luciferase) of pCX-Luc2 was inserted into a lentiviral vector (pCDH, System Biosciences, Palo Alto, CA) to construct pCDH-CAG-Luc2. Lentiviral particles were prepared according to a protocol with minor modifications [14]. Briefly, 2×10^7^ of 293FT cells (Thermo Fisher) were seeded on four 10 cm-dishes, and cultured overnight. Cells were transfected with DNA of 40 μg pCDH-CAG-Luc2 plasmid, 26 μg psPAX2 packaging plasmid, and 14 μg pMD2.G envelope plasmid, with the use of Lipofectamine 2000 (Thermo Fisher). Viral particles were harvested on the second and third days after transfection, and precipitated using PEG-it Virus-Precipitation Solution (System Biosciences). Viral particles were resuspended in 2 ml of the washing medium, aliquoted into forty 1.5 ml-tubes (50 μl each), and stored at –80°C. Spheroids cultured in one well of a 12-well cell-culture plate were trypsinized, and about half of the cells were collected by centrifugation (200 × *g*) in a 1.5 ml-tube. Cells were resuspended in 50 μl of the virus solution containing 10 μg/ml hexadimethrine bromide and 10 μM Y27632, and incubated at 37 °C for 6 h. Then, the cells were collected by centrifugation (200 ×*g*), resuspended in 60 μl of Matrigel, and seeded into two wells of a 12-well cell-culture plate.

### Luminescence-based growth monitoring in spheroid culture

For growth monitoring assays, luciferase-expressing spheroids were cultured in 2–3 wells of a 12-well cell-culture plate. Spheroids were trypsinized, filtered through a 40-μm cell strainer (Corning), and resuspended in 300 μl of Matrigel for a 96-well plate. Dilution (based on the volume of Matrigel) was adjusted 4–8 times depending on the growth rate and spheroid density. The spheroid cells in Matrigel were distributed to the wells of 96-well “white” cell-culture plates (Corning; 3 μl per well) on a plate heater at 37°C, and cultured overnight in 100 μl/well of the medium. Spheroids were rinsed with 100 μl/well of PBS, and incubated with 50 μl/well of 150 μg/ml luciferin in the phenol red-free DMEM/F12 medium (Nacalai) at room temperature (20–28 °C) for 10 min. Luminescence was scored using a conventional imaging device (Gel Doc XR+, BioRad, Hercules, CA). After this initial measurement, spheroids in each well were rinsed with 100 μl of PBS, and cultured in 100 μl of the selected medium. Luminescence was determined daily or 3 days later. The cell growth rate for each well was estimated as the proportion of photon counts to those on the initial measurement. In Y27632 treatment experiments, the initial and second measurements were performed on post-passage days 0 and 2, respectively. Three to six replicates were analyzed for each data point in all experiments except for the chemical compound screening assays (resulted in similar statistical power).

### Preparation of RNA from spheroids

After aspiration of the medium, the lysis buffer (Takara Bio Inc., Kusatsu, Japan) was directly added to each well of spheroid culture. RNA was purified using NucleoSpin RNA II kit (Takara).

### Quantitative RT-PCR (qRT-PCR)

Molecules of cDNA were synthesized using SuperScript III (Thermo Fisher), and qPCRs were performed using SYBR Green reagents (Toyobo, Osaka, Japan) in an ABI StepOnePlus thermal cycler (Thermo Fisher). Expression levels were normalized relative to those of *ACTB*. Sequences of primer pairs were as follows: *AXIN2*, CATGACGGACAGCAGTGTAGA and AACTCCAGCTTCAGCTTTTCC; *MKI67*, AAGAGAGTGTCTATCAGCCGAAGT and GTGGCCTGTACTAAATTGACTGTG; *ACTB*, CATGTACGTTGCTATCCAGGC and CTCCTTAATGTCACGCACGAT.

### Preparation of DNA and histology specimens from spheroids

Spheroids in Matrigel were suspended in Cell Recovery Solution (Corning), and collected in 1.5 ml-tubes. Matrigel was digested at 4 °C with rotation for 30–60 min. Spheroids were centrifuged at 200 ×*g* for 5 min, and washed with PBS twice at 4 °C. DNA was purified using DNeasy Blood & Tissue Kit (Qiagen, Hilden, Germany). For histological analyses, spheroids were passaged once or twice without trypsinization so that larger cell aggregates were formed. They were embedded in iPGell (Genostaff, Tokyo, Japan), and fixed with 4% paraformaldehyde in PBS at 4°C for 16 h.

### Histopathological classification of colorectal cancer

Formalin-fixed paraffin-embedded specimens were sectioned at 4-μm thickness, and stained with H&E. Histological images were captured through Leica DM2000 microscope (Leica Microsystems, Wetzlar, Germany) equipped with OLYMPUS DP73 digital camera and cellSens Standard 1.6 software (OLYMPUS). Histological grades of primary cancer and spheroids were determined according to the WHO guideline and the recommendation of the College of American Pathologists [16, 17].

### Mutational hotspot analysis

Hotspot mutations in 50 cancer-related genes were detected by Macrogen (Seoul, Republic of Korea) using Ion AmpliSeq Cancer Hotspot Panel v2 (Thermo Fisher). The list of sequenced genes and mutations is available from manufacturer’s website (http://tools.invitrogen.com/downloads/cms_106003.csv). The data sets were analyzed using Integrative Genomics Viewer software (Broad Institute).

### Array-based comparative genomic hybridization (CGH) analysis

Array-based CGH analyses were performed with Agilent SurePrint G3 human CGH microarray 1×1M (G4447A, Agilent, Santa Clara, CA) and Genomic DNA ULS Labeling Kit (#5190-0419, Agilent) according to the manufacturer’s instructions. Human Genomic DNA (G1521 and G1471, Promega) was used as the control. The array slides were scanned using Agilent G2565BA microarray scanner (Agilent) at the Medical Research Support Center, Graduate School of Medicine, Kyoto University.

### Statistical tests

All statistical analyses were performed using GraphPad PRISM software version 6 (GraphPad Software, La Jolla, CA).

### Dose-response curve

The luminescence signal intensities were normalized and fitted to the dose-response model using PRISM version 6.

## SUPPLEMENTARY MATERIALS FIGURES



## Abbreviations

(PD-CRC): Patient-derived colorectal cancer
(TIC): tumor initiating cell
(EGF): epidermal growth factor
(bFGF): basic fibroblast growth factor
(NECA): 5′-(*N*-ethyl-carboxamido)-adenosine
(EGFR): epidermal growth factor receptor
(VEGF): vascular endothelial growth factor
(PDX): patient-derived xenograft
(ROCK): Rho-associated coiled-coil protein kinase
(TGF-β): transforming growth factor β
(SC): stem cell
(GEI): growth effect index
(PPAR): peroxisome proliferator-activated receptor
(AR): adenosine receptor
(KCa): calcium-activated potassium-channel
(cAMP): cyclic AMP
(PKA): protein kinase A
(MAPK): mitogen-activated protein kinases
(ERK): extracellular signal-regulated kinases
(PDSX): patient-derived spheroid xenografts
