# Supplementary material for: An improved method for culturing patient-derived colorectal cancer spheroids
Source: Oncotarget. 2018 Apr 24;9(31):21950–64. doi: 10.18632/oncotarget.25134 (PMC5955161; doi:10.18632/oncotarget.25134)
Supplement: Supplementary file 1 [file oncotarget-09-21950-s001.pdf]

An improved method for culturing patient-derived colorectal cancer spheroids

SUPPLEMENTARY MATERIALS

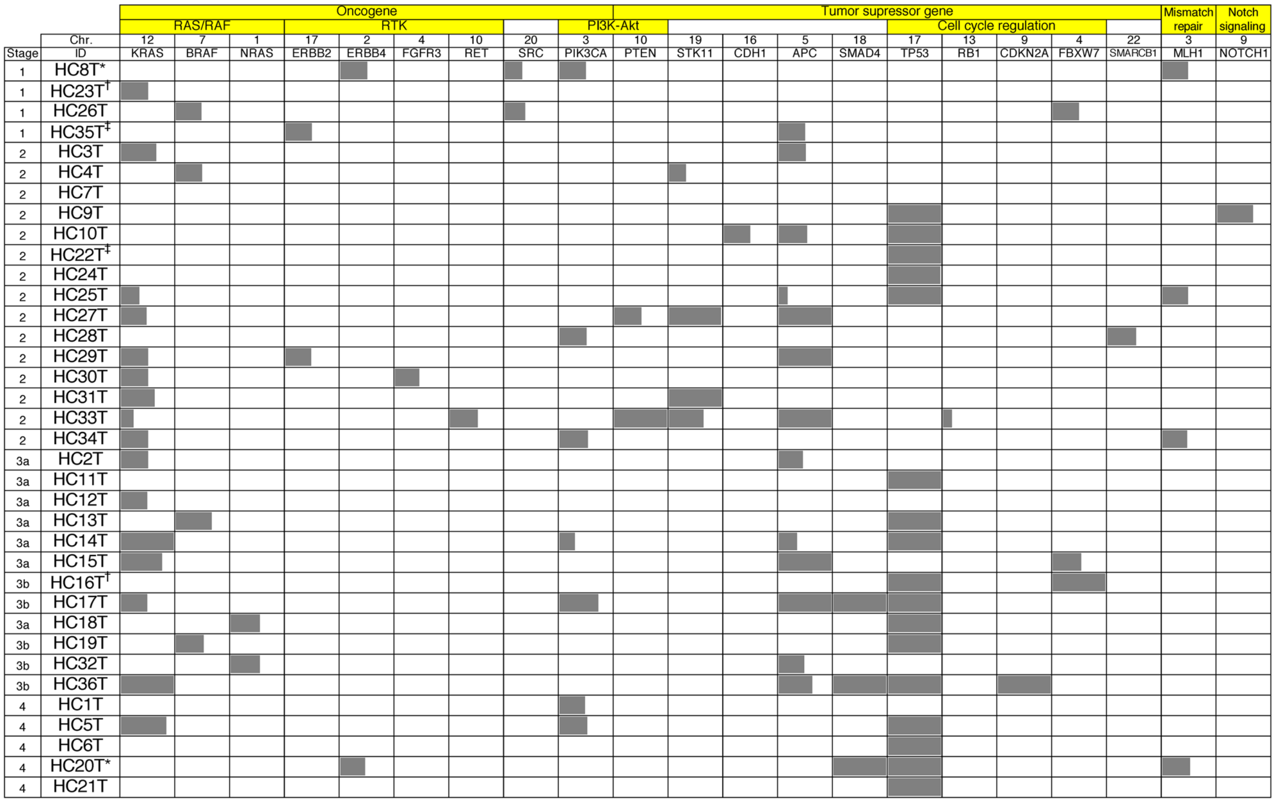

Supplementary Figure 1: Mutations in 21 oncogenes and tumor suppressor genes of 36 CRC-TIC spheroid lines. The genomic regions covered by a hotspot gene panel (see MATERIALS AND METHODS) were sequenced. Shown are mutations that affect the amino acid sequences of the encoded proteins. Genes were classified into functional categories shown at the top. A gray rectangle in each cell represents a heterozygous (partially filled) or homozygous (full-filled) mutation. *, †, ‡, Spheroid lines labeled with the same symbols derived from the same patients. Note that only 50.5% of mutations in the *APC* gene was detectable due to limited coverage of the panel (based on the COSMIC database; http://cancer.sanger.ac.uk/cosmic).

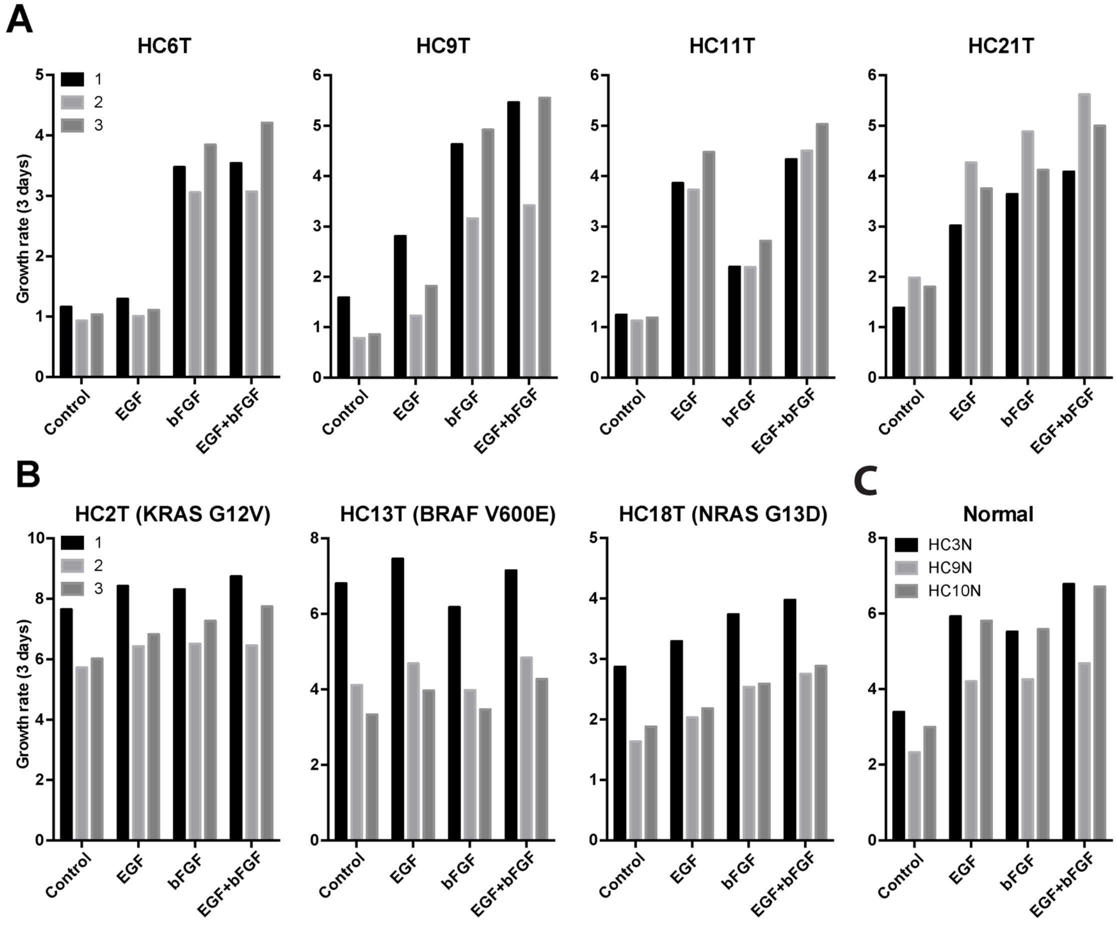

Supplementary Figure 2: Growth-promoting effects by EGF and/or bFGF. Spheroids of CRC-TICs and normal colonic epithelial SCs were treated with EGF and/or bFGF throughout post-passage days 1–4. Growth rates of the luciferase-expressing spheroids were calculated from their bioluminescence levels on post-passage days 1 and 4. **(A)** Growth rates of CRC-TIC lines carrying the wild-type *RAS*/*RAF* genes. **(B)** Growth rates of CRC-TIC lines carrying mutant *RAS*/*RAF* genes. **(C)** Growth rates of normal colonic epithelial spheroids cultured in the eL-WRN medium. Black and gray bars show the mean growth rates of six replicates of three independent experiments in (A) and (B), whereas those in (C) represent three independent spheroid lines as indicated.

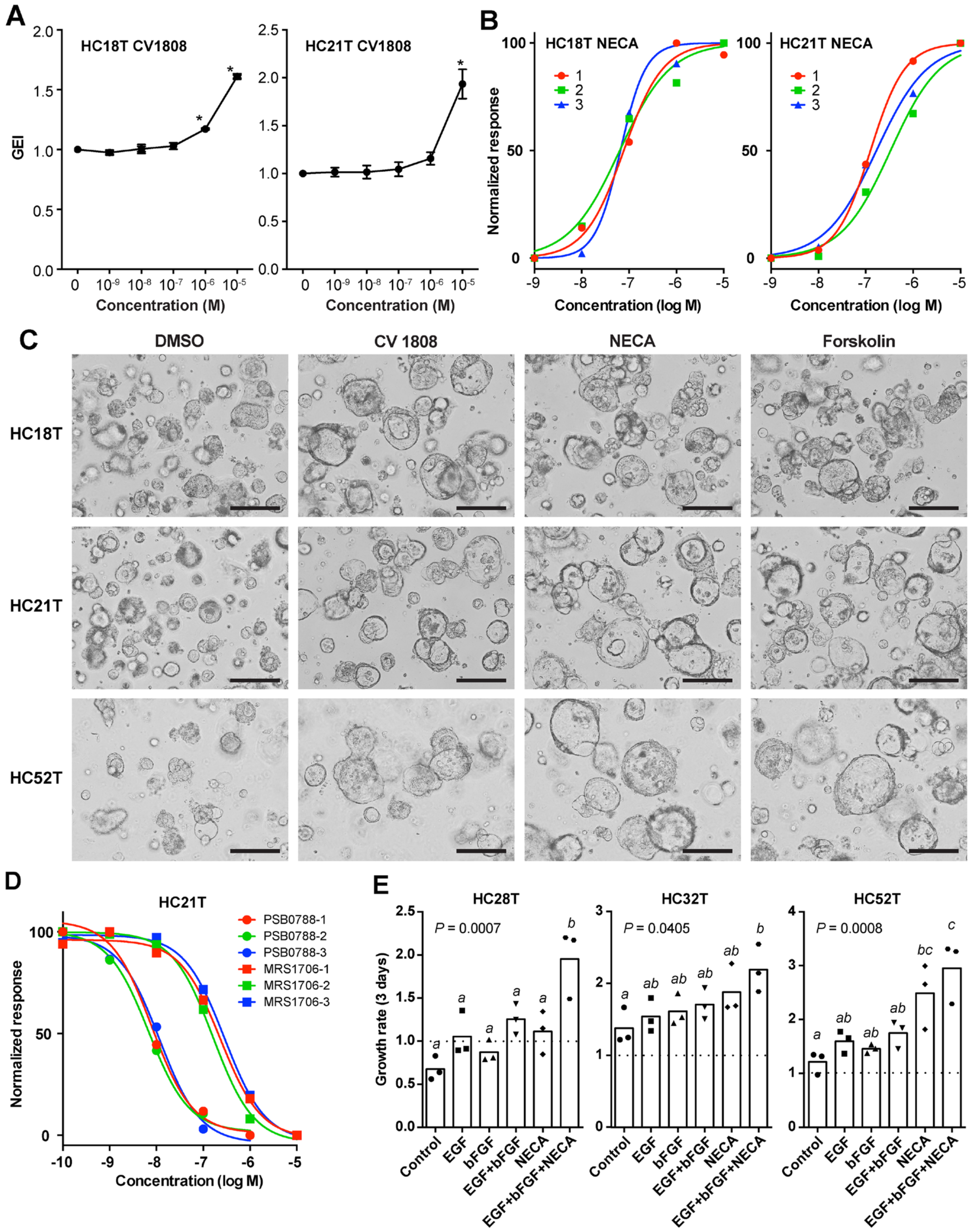

Supplementary Figure 3: Effects of adenosine receptor agonists on spheroid culture. **(A)** Dose-dependent effects of CV1808 on the growths of two colorectal cancer spheroid lines (HC18T and HC21T). Shown are the mean GEI ± SD (independent experiments, n = 3). Asterisks indicate statistically significant differences from the solvent-only (0 μM) spheroids ($P < 0.05$; analyzed using one-way ANOVA followed by Tukey's post-test). **(B)** Reproducibility of the NECA dose-response curves in repeated experiments. The same data sets in Figure 6B were used to perform curve-fitting analyses for the NECA treatment. Shown are fitted curves of the normalized data in three independent experiments. **(C)** Representative phase-contrast micrographs of CRC-TIC spheroids after treatments with cAMP-signaling activators. Colorectal cancer spheroids (HC18T, HC21T, and HC52T) were cultured with solvent only (DMSO), 10 μM CV1808, 1 μM NECA, or 1 μM forskolin for five days. Scale bar, 200 μm. **(D)** Reproducibility of the dose-response curves for A_{2B} inhibitors in repeated experiments. The same data sets in Figure 6D were used to perform the curve-fitting analysis for each A_{2B} inhibitor treatment. **(E)** Growth stimulation by three supplementary factors on the slow-growing spheroid lines. Luciferase-expressing spheroids were cultured in the presence of the indicated factor(s) for 3 days. Plotted are the growth rates with means in three independent experiments. Data were analyzed using one-way ANOVA (P values are shown in the graphs) followed by Tukey's post-test. The mean values between the different letters are statistically different ($P < 0.05$). Note that growth rates in the control spheroids are ~1.0 (dotted lines), which implies their difficulty in growth.

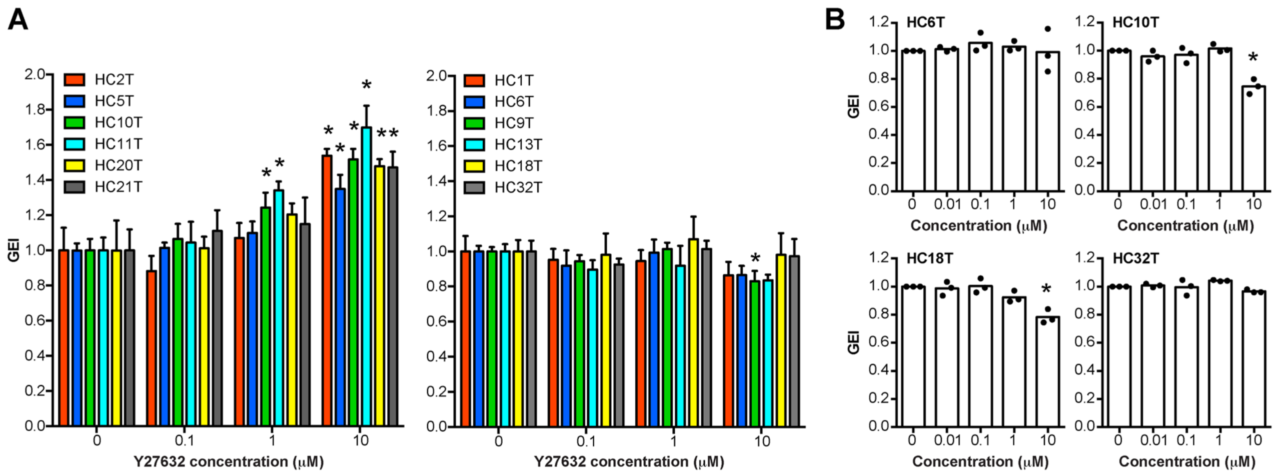

Supplementary Figure 4: Optimization of the culture media. (A) Dose-dependent effects of Y27632 on the growths of CRC spheroid lines. Luciferase-expressing cancer spheroids were cultured for two days in the EGF/bFGF-containing cancer medium in the presence of Y27632 at the indicated concentrations. Tested were twelve CRC spheroid lines showing (*left*) or not showing (*right*) growth-promoting effects of Y27632. Shown are the mean GEI + SD (three or four replicated wells). Asterisks indicate statistically significant differences from the solvent-only (0 μM) spheroids ($P < 0.05$; analyzed using one-way ANOVA followed by Tukey's post-test). **(B)** Dose-dependent effects of SB431542 on the growths of cancer spheroid lines (continued from Figure 7A). Plotted are the GEI with means in three independent experiments. Asterisks indicate statistically significant differences from the solvent-only (0 μM) spheroids ($P < 0.05$; analyzed using one-way ANOVA followed by Tukey's post-test).
